# From wernicke-korsakoff to central pontine myelinolysis: the potentially irreversible risks of alcohol use

**DOI:** 10.1192/j.eurpsy.2024.820

**Published:** 2024-08-27

**Authors:** A. F. Silva, R. M. Lopes, V. S. Melo, C. A. Rodrigues, P. M. Coelho, F. M. A. Santos, I. S. Fernandes, L. P. Delgado

**Affiliations:** Psychiatry and Mental Health Department, Centro Hospitalar Médio Tejo, Tomar, Portugal

## Abstract

**Introduction:**

Sustained alcohol intake, when combined with incomplete treatment, can result in chronic structural changes in the Central Nervous System, including generalized cortical and cerebellar atrophy, amnesic syndromes like Korsakoff’s syndrome, and white matter disorders such as Central Pontine Myelinolysis and Marchiafava-Bignami syndrome. It is crucial to prevent these complications due to their potential for irreversible and debilitating consequences. For Wernicke-Korsakoff syndrome, early recognition and thiamine administration for prevention are paramount, as it arises from thiamine deficiency due to malnutrition caused by persistent alcohol use. In the case of Central Pontine Myelinolysis, which is caused by abrupt fluctuations in serum osmolality, controlled sodium correction is essential.

**Objectives:**

Through a clinical case and a review of published literature, this study aims to reflect on the importance of preventing neurological injuries associated with chronic alcohol consumption, specifically Wernicke-Korsakoff Syndrome and Central Pontine Myelinolysis.

**Methods:**

A literature review was conducted by searching for articles on PubMed using the terms “Alcohol Use Disorder,” “Wernicke-Korsakoff syndrome,” and “Central pontine myelinolysis.” A clinical case is presented, featuring a 50-year-old patient with alcohol use disorder who developed Wernicke-Korsakoff syndrome and Central Pontine Myelinolysis. Considering this case, we reflect on the primary approaches that could have been beneficial in preventing these complications and propose a straightforward method for doing so.

**Results:**

A 50-year-old patient presented with poor general condition, characterized by low weight, significant loss of strength in the limbs and arms, and incoherent speech with anterograde amnesia and confabulation. This condition had progressed to a point where the patient could no longer walk, perform basic self-care tasks such as bathing, dressing, and eating independently, underscoring the severity of his condition. The diagnoses of Wernicke-Korsakoff syndrome and Central Pontine Myelinolysis were established based on clinical manifestations and the presence of hyperintense lesions observed in the central pons on T2/FLAIR axial MRI scans. This clinical case highlights the importance of proper and precocious prevention of complications in patients with alcohol use disorder. The foremost step in preventing these complications is to treat alcohol dependence effectively, even when faced with patient resistance. It’s vital to remain vigilant about potential complications and implement suitable prophylactic measures.

**Conclusions:**

The devastating effects of complications arising from Alcohol Use Disorder, such as Wernicke-Korsakoff syndrome and Central Pontine Myelinolysis, underscore the importance of enhanced attention that clinicians should provide when approaching these patients at all clinical interactions.

**Disclosure of Interest:**

None Declared

